# Skin Advanced Glycation End Products among Subjects with Type 2 Diabetes Mellitus with or without Distal Sensorimotor Polyneuropathy

**DOI:** 10.1155/2021/6045677

**Published:** 2021-11-28

**Authors:** Stella Papachristou, Kalliopi Pafili, Grigorios Trypsianis, Dimitrios Papazoglou, Konstantinos Vadikolias, Nikolaos Papanas

**Affiliations:** ^1^Diabetes Centre-Diabetic Foot Clinic, Second Department of Internal Medicine, Democritus University of Thrace, University Hospital of Alexandroupolis, Greece; ^2^Department of Medical Statistics, Medical School, Democritus University of Thrace, Alexandroupolis, Greece; ^3^Department of Neurology, Democritus University of Thrace, University Hospital of Alexandroupolis, Greece

## Abstract

**Materials and Methods:**

We included 132 subjects (88 men) with a mean age of 64.57 years and median T2DM duration of 14.5 years. Skin AGEs were measured with AGE reader mu connect (Diagnoptics) on the dominant arm. The device enables single and automated triplicate measurements: both of these were performed. DSPN was diagnosed through the neuropathy disability score (NDS). Small nerve fibre function was assessed by temperature and pinprick sensation on the foot. Bilateral measurement of the vibration perception threshold (VPT) on the hallux was carried out by using a neurothesiometer (Horwell Scientific Laboratory Supplies).

**Results:**

Single and triplicate AGE measurements were positively correlated with each other (Pearson's correlation coefficient *r* = 0.991, 95%CI = 0.987-0.994, *p* < 0.001). AGEs were higher among subjects with vs. those without DSPN (*p* < 0.001). Furthermore, they were higher among subjects with reduced vs. normal temperature sensation (*p* < 0.001), among subjects with reduced vs. normal pinprick sensation (*p* = 0.002), among those with abnormal vs. normal monofilament examination (*p* < 0.001), and among those with abnormal vs. normal VPT (*p* < 0.001). AGEs were correlated with NDS, VPT, and monofilament score.

**Conclusions:**

In T2DM, skin AGEs are increased in the presence of DSPN. This holds true both for large and for small nerve function impairment. Moreover, AGEs are correlated with DSPN severity.

## 1. Introduction

Diabetic neuropathy remains a major chronic complication of diabetes mellitus [1]. It may, in turn, lead to further complications, notably diabetic foot, neuropathic pain, and autonomic failure [2]. Its commonest manifestation is chronic distal sensorimotor polyneuropathy (DSPN) [3]. Chronic hyperglycaemia represents a major underlying pathogenic mechanism [4]. Excess serum glucose activates several biochemical pathways and, among others, leads to the formation of advanced glycation end products (AGEs) [5, 6]. The latter promote inflammation and impair normal electrical activity in neurones [6]. AGEs may be measured by the enzyme-linked immunosorbent assay (ELISA), high-performance liquid chromatography (HPLC), mass spectrography, and tissue biopsy or through evaluation of skin autofluorescence [5, 6]. In recent years, their measurement in the skin has attracted considerable interest, because it is noninvasive and accurate [5–7]. It has been discussed that skin AGEs measurement might serve as a risk marker of atherosclerotic disease [7, 8] or of microvascular complications of diabetes [6, 9].

However, the association of AGEs with DSPN is still not demonstrated enough. Therefore, the aim of this study was to examine the correlation between skin AGEs and parameters of DSPN in subjects with type 2 diabetes mellitus (T2DM).

## 2. Materials and Methods

This study included 132 subjects (88 men, 44 women) with a mean age of 64.57 ± 8.21 years and median T2DM duration of 14.5 years (range 7.00-20.00) who were attending the Diabetes Centre of the Second Department of Internal Medicine at Democritus University of Thrace, Greece. These were randomly chosen and offered an examination. The study was approved by the institutional ethics committee, and all patients gave their informed consent.

Inclusion criteria were age above 18 years and T2DM. Exclusion criteria were as follows: age ≥ 85 years, inability to undertake the examination, severe illness, severe infection, hypoglycaemia, liver cirrhosis, alcohol abuse, B12 depletion, other causes of neuropathy, heart failure, dermatologic disease at the measuring site, tattoos, exposure to skincare creams or any other substance that may have fluorescent properties, self-tanning agents in the past 10 days, Fitzpatrick skin type > V, and chronic kidney disease (estimated glomerular filtration rate < 60 mg/dl) [6, 7].

Skin AGEs were measured with AGE reader mu connect (Diagnoptics, NL) on the dominant arm, according to the manufacturer's instructions [4]. Subjects were asked to place their dominant forearm on the device, the elbow being aligned with the reader's edge. Measurements were taken once the dominant arm was in the correct position. The device illuminates a small portion of the skin (approximately 4 cm^2^) on the volar side of the examinee's forearm [4]. It produces light on the selected area with an excitation light source of ∼370 nm. Emission light and reflected excitation light emanating from the skin are measured using a glass fibre in the 300-600 nm range [4]. The device enables single and automated triplicate measurements: both of these were performed [4]. AGEs were expressed in arbitrary units, as per the manufacturer [4].

Diagnosis of DSPN was based on the neuropathy disability score (NDS), an established clinical examination score [10]. DSPN was defined as NDS ≥ 3 [10]. In a simplified approach based on its original classification [10], DSPN was defined as absent (NDS 0-2), mild (NDS 3-5), and moderate/severe (NDS 6-10).

Small nerve fibre function was evaluated by temperature and pinprick sensation on the foot [11]. These were evaluated on the dorsal foot aspect using a Tiptherm rod and a sterile single-use lancet, respectively [11].

The vibration perception threshold (VPT) on the hallux was measured bilaterally with a neurothesiometer (Horwell Scientific Laboratory Supplies) [12]. Abnormality was defined as VPT > 25 V, using the lower of the two measurements [12]. Then, patients were classified into those with normal (<16 V), mildly impaired (16-25 V), and severely impaired VPT (>25 V) [13, 14].

Finally, 10 g Semmes Weinstein monofilaments were used on 10 foot sites [14, 15] bilaterally. The monofilament score was the number of correct answers [14]. Abnormality was defined as monofilament score < 8, using the lower of the two measurements [14, 15].

### 2.1. Statistical Analysis

Analysis was carried out using the IBM Statistical Package for the Social Sciences (SPSS), version 19.0 (IBM Corp., Armonk, NY, USA). The normality of quantitative variables was tested by the Kolmogorov-Smirnov test. Normally distributed quantitative variables were expressed as mean ± standard deviation (SD), while qualitative variables were expressed as absolute and relative (%) frequencies. The association of AGEs with patients' demographic and clinical characteristics was assessed using Student's *t*-test and analysis of variance (ANOVA); post hoc comparisons were performed using Tukey's test. Correlations were assessed by Pearson's *r* and intraclass (ICC, two-way mixed effects model with average measures) correlation coefficients.

Receiver operating characteristic (ROC) analysis was used to evaluate the diagnostic significance of single AGEs measurement and triplicate AGEs measurement for large fibre impairment (impaired tuning fork perception or ankle reflexes), small fibre impairment (impaired temperature or pinprick sensation), and overall DSPN. The area under the ROC curve (AUC), sensitivity, specificity, and positive and negative predictive values were calculated, while Cohen's kappa was used to assess agreement. The optimal cut-off values were derived according to the Youden index [16]. All tests were two-tailed. Statistical significance was defined at 5% (*p* < 0.05).

## 3. Results

Single AGEs measurement was positively correlated with their triplicate measurement (Pearson's correlation coefficient *r* = 0.991, 95%confidence interval (CI) = 0.987-0.994, *p* < 0.001; intraclass correlation coefficient (ICC) = 0.995, 95%CI = 0.994-0.997, *p* < 0.001).

In single measurement, AGEs were higher among subjects with vs. those without DSPN (3.31 ± 0.73 vs. 2.55 ± 0.56, *p* < 0.001). Furthermore, they were higher among subjects with reduced vs. normal temperature sensation (*p* < 0.001), among subjects with reduced vs. normal pinprick sensation (*p* = 0.002), among those with abnormal vs. normal VPT (*p* < 0.001), and among those with abnormal vs. normal monofilament examination (*p* < 0.001) (Table 1). Identical significant differences were observed in triplicate measurement (data not shown).

AGEs (single measurement) showed positive correlations with age (*r* = 0.343, *p* < 0.001), T2DM duration (*r* = 0.275, *p* = 0.001), NDS (*r* = 0.551, *p* < 0.001), VPT right foot (*r* = 0.475, *p* < 0.001), VPT left foot (*r* = 0.422, *p* < 0.001), monofilament score right foot (*r* = −0.462, *p* < 0.001), and monofilament score left foot (*r* = −0.484, *p* < 0.001). Similarly, AGEs (triple measurement) showed positive correlations with age (*r* = 0.361, *p* < 0.001), T2DM duration (*r* = 0.283, *p* = 0.001), NDS (*r* = 0.555, *p* < 0.001), VPT right foot (*r* = 0.482, *p* < 0.001), VPT left foot (*r* = 0.422, *p* < 0.001), monofilament score right foot (*r* = −0.472, *p* < 0.001), and monofilament score left foot (*r* = −0.482, *p* < 0.001).

AGEs in relation to the severity of DSPN (evaluated by NDS and VPT) are shown in Table 2. AGEs (both single and triplicate measurements) were significantly higher among subjects with severe impairments.

**Figure 1:**
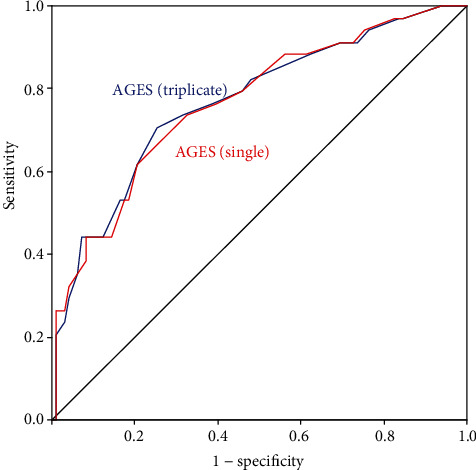
ROC analysis for the evaluation of the diagnostic significance of AGEs (single measurement) and AGEs (triplicate measurement) for large fibre impairment.

**Figure 2:**
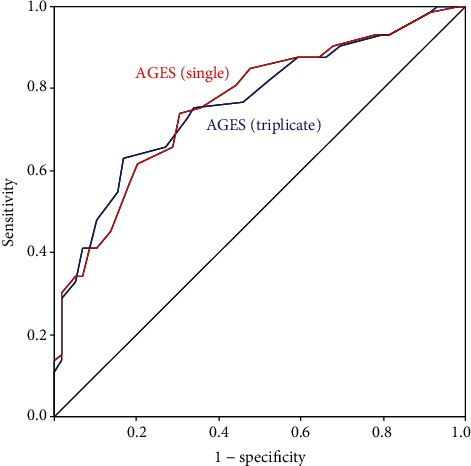
ROC analysis for the evaluation of the diagnostic significance of AGEs (single measurement) and AGEs (triplicate measurement) for small fibre impairment.

**Figure 3:**
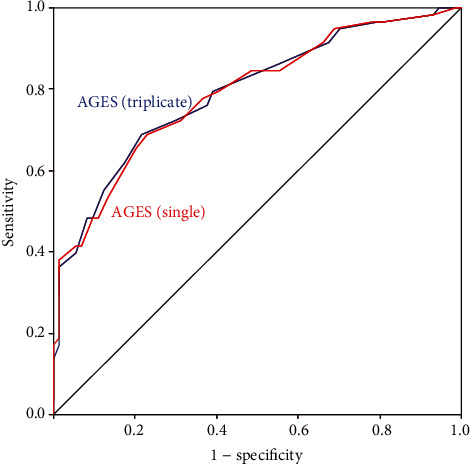
ROC analysis for the evaluation of the diagnostic significance of AGEs (single measurement) and AGEs (triplicate measurement) for overall DSPN.

Table 3 summarises ROC analysis for the evaluation of the diagnostic significance of AGEs (single measurement) for large fibre impairment, small fibre impairment, and overall DSPN. The optimal cut-offs were ≥3.15 for large fibre impairment, ≥2.75 for small fibre impairment, and ≥2.95 for overall DSPN. The corresponding AUC values (and 95% confidence intervals) were 0.766 (0.671-0.861), 0.765 (0.685-0.846), and 0.790 (0.712-0.869) (on each occasion, *p* < 0.001). With these cut-offs, AGEs (single measurement) yielded moderately high agreement (>71%), sensitivity (>61%), specificity (>69%), and negative predictive value (NPV) (>68%), as well as low-moderate positive predictive value (PPV) (>51%). The highest sensitivity and PPV were seen for small fibre impairment, while the highest specificity and NPV were seen for large fibre impairment. ROC analysis was the same for triplicate AGE measurements (data not shown). ROC curves for both single and triplicate AGE measurements are shown in Figures 1–3.

## 4. Discussion

In T2DM, the present study has shown that skin AGEs are increased in the presence of DSPN. This holds true both for large and for small nerve fibre function impairment. Moreover, a positive correlation between skin AGEs and DSPN severity was noted.

Our findings are in line with previous reports [17–22]. Indeed, in a multicentre study including 497 participants with diabetes mellitus (including both diabetes types), a significant increase in skin AGEs was seen in the presence of DSPN (defined using the Toronto Clinical Neuropathy Score, the Neuropathy Symptom Score, and the NDS) [17]. Skin AGEs were elevated among participants with NDS ≥ 3, compared with those exhibiting a lower NDS score [17]. Further evidence pointed to an increased accumulation of skin AGEs in T2DM Japanese subjects with DSPN (diagnosed by the presence of ≥1 neuropathic symptoms, abnormal vibration perception, and absence of ankle/knee reflexes), as compared with those without [18, 19]. When DSPN was defined as history of diabetic foot ulceration, a positive correlation was seen with the higher tertiles of skin AGEs, even following adjustment for several confounding factors including macrovascular disease [20].

A more recent work including 820 T2DM Chinese participants used nerve conduction study for DSPN diagnosis [21]. Higher skin AGEs were linked with a five-fold increased risk of DSPN (odds ratio = 5.15; 95%CI = 1.48-4.53, *p* < 0.01), and a cut-off value > 2.57 predicted a threefold increased risk of DSPN [21]. Taken together, our and previous reports reveal an association between elevated skin AGEs and both DSPN presence and severity. Of note, the latter has been diagnosed by various modalities.

Importantly, the present study appears to be the only one to have looked separately at large and small fibre impairment. Indeed, skin AGEs were increased in both small and large nerve fibre impairments. As regards the former, reduced temperature and pinprick sensation (estimates of small nerve fibres [14, 15]) were seen in participants with higher skin AGE levels. The same was seen for parameters assessing the latter (abnormal VPT and monofilament). So far, a positive association between skin AGEs and increased VPT, even before reaching the VPT cut-off indicating high risk of foot ulceration, has been reported [23, 24]. Similarly, nerve conduction velocity and amplitude of the median, sural, and peroneal nerve were negatively correlated with skin AGEs among diabetic participants (both diabetes types) with or without DSPN [25].

As regards small nerve fibres, our findings are in line with the previously reported inverse relationship between skin AGEs and electrochemical skin conductance [26]. However, we assessed small fibre impairment differently from the previous study.

Skin AGEs (either single or triple measurement) in our study showed positive correlations with age, T2DM duration, NDS, VPT, and monofilament score in each lower extremity. Again, these results are in line with previous reported associations of AGEs with age, smoking, renal function, macroangiopathy, and microvascular complications [19, 27]. Other associations have previously included body mass index, HbA_1c_, high-density lipoprotein cholesterol, and albumin-to-creatinine ratio [28]. Interestingly, in a study registering the most ancient available previous HbA_1c_ before admission to the study [20], skin autofluorescence was independently related to this and not the most recent HbA_1c_, potentially demonstrating glucose memory as one of the main variables for skin AGEs accumulation.

Triplicate measurements were highly correlated with single measurements. All results and associations observed with single measurements were also observed with triplicate measurements with almost identical numbers. This finding is novel and suggests that triplicate AGEs measurements are not required to study the association with DSPN. This holds true, although triplicate measurements are also simple and quick to perform.

Interestingly, with appropriate cut-offs identified by ROC analysis, AGEs measurement (both single and triplicate) yielded moderately high sensitivity, specificity, and NPV, while its PPV was low-moderate. This held true for large fibre impairment, small fibre impairment, and overall DSPN. The highest sensitivity and PPV were seen for small fibre impairment, while the highest specificity and NPV were seen for large fibre impairment. Based on these observations, the contribution of AGEs measurement to DSPN diagnosis is rather moderate. Arguably, they may be slightly more useful for the diagnosis of small fibre impairment and for the exclusion of large fibre impairment, but caution and further experience are needed.

The strength of the study is the inclusion of several DSPN parameters. A limitation is the smaller number of female than male participants. A second limitation is the clinical diagnosis of DSPN. Indeed, we did not use more sophisticated modalities, such as nerve conduction velocity, intraepidermal nerve fibre density via skin biopsy, or corneal confocal microscopy, which may enable earlier diagnosis of subclinical DSPN [1, 2, 29]. Moreover, subjects were included in a tertiary care centre, and so results may not be directly applicable to primary care and/or the general T2DM population. Furthermore, our study offers no prospective data on DSPN development, but this was beyond its scope. Finally, extending our observations to other microvascular complications (microalbuminuria, diabetic retinopathy) would be interesting, but this was beyond the scope of this work focusing on neuropathy.

The implications of the present study may be outlined as follows. Skin AGEs are higher in the presence of DSPN and are associated with its severity. Whether they represent an aetiological factor or whether they are merely a manifestation of nerve damage cannot be answered by the present study. Indeed, longitudinal data looking at the risk of developing DSPN in relation to AGEs would be required to clarify this issue. We also need more information as to how skin AGEs might, perhaps, be used as a screening tool of DSPN in everyday practice. For this purpose, simplicity and rapidity of the examination would be useful advantages, but the cost and limited availability of the device are important disadvantages.

## 5. Conclusions

In T2DM, skin AGEs are increased in the presence of DSPN. This holds true both for large and for small nerve function impairment, as well as for loss of protective sensation. Moreover, AGEs are correlated with DSPN severity. These results add to our insights into the role of AGES in DSPN and suggest that their further study including prospective data is justified.

## Figures and Tables

**Table 1:** AGEs (single measurement) and parameters of DSPN.

AGEs (single measurement)			
Parameter	With DSPN	Without DSPN	*p* value
	3.31 ± 0.73	2.55 ± 0.56	<0.001
	Reduced temperature sensation	Normal temperature sensation	*p* value
	3.18 ± 0.72	2.51 ± 0.56	<0.001
	Reduced pinprick sensation	Normal pinprick sensation	*p* value
	3.86 ± 0.32	2.84 ± 0.72	0.002
	Abnormal VPT	Normal VPT	*p* value
	3.45 ± 0.69	2.74 ± 0.68	<0.001
	Abnormal monofilament	Normal monofilament	*p* value
	3.19 ± 0.69	2.62 ± 0.67	<0.001

AGEs: advanced glycation end products; DSPN: distal sensorimotor polyneuropathy; VPT: vibration perception threshold.

**Table 2:** AGEs in relation to the severity of DSPN.

	AGEs (single)	*p* value	AGEs (triplicate)	*p* value
NDS		<0.001		<0.001
No DSPN (*n* = 76)	2.55 ± 0.55		2.55 ± 0.54	
Mild DSPN (*n* = 43)	3.27 ± 0.74		3.25 ± 0.71	
Moderate/severe DSPN (*n* = 13)	3.50 ± 0.69		3.47 ± 0.71	
Multiple comparisons				
No DSPN vs. mild DSPN	—	<0.001	—	<0.001
No DSPN vs. moderate/severe DSPN	—	<0.001	—	<0.001
Mild DSPN vs. moderate/severe DSPN	—	0.500	—	0.484
VPT		<0.001		<0.001
Normal (*n* = 62)	2.58 ± 0.58		2.56 ± 0.56	
Mildly impaired (*n* = 54)	3.02 ± 0.77		3.00 ± 0.74	
Severely impaired (*n* = 16)	3.58 ± 0.56		3.59 ± 0.58	
Multiple comparisons				
Normal vs. mildly impaired	—	0.001	—	0.001
Normal vs. severely impaired	—	<0.001	—	<0.001
Mildly impaired vs. severely impaired	—	0.010	—	0.005

AGEs: advanced glycation end products; DSPN: distal sensorimotor polyneuropathy; NDS: neuropathy disability score; VPT: vibration perception threshold.

**Table 3:** ROC analysis for the evaluation of the diagnostic significance of AGEs (single measurement) for large fibre impairment, small fibre impairment, and overall DSPN.

	Large fibre	*p* value	Small fibre	*p* value	Overall DSPN	*p* value
AUC (95% CI)	0.766 (0.671-0.861)	<0.001	0.765 (0.685-0.846)	<0.001	0.790 (0.712-0.869)	<0.001
Cut-off	≥3.15		≥2.75		≥2.95	
Sensitivity (%)	61.8 (43.6-77.8)		74.0 (62.4-83.6)		69.0 (55.5-80.5)	
Specificity (%)	79.6 (70.3-87.1)		69.5 (56.1-80.8)		77.0 (65.8-86.0)	
PPV (%)	51.2 (39.6-62.7)		75.0 (66.6-81.9)		70.2 (60.0-78.7)	
NPV (%)	85.7 (79.5-90.3)		68.3 (58.6-76.7)		76.0 (67.9-82.6)	
Overall agreement (%)	75.0		72.0		73.5	
Cohen's kappa	0.388	<0.001	0.434	<0.001	0.461	<0.001
OR (95% CI)	6.30 (2.70-14.72)	<0.001	6.47 (3.02-13.87)	<0.001	7.45 (3.43-16.20)	<0.001

AUC: area under the curve; CI: confidence interval; DSPN: distal sensorimotor polyneuropathy; NPV: negative predictive value; OR: odds ratio; PPV: positive predictive value.

## Data Availability

Data is available upon reasonable request.
